# The sORF-Encoded Peptides, ATP Synthase Subunits, Facilitate WSSV Duplication in Shrimp

**DOI:** 10.3390/v14112449

**Published:** 2022-11-04

**Authors:** Li-Jie Huo, Peng-Yuan Lu, Dian-Xiang Li, Xiu-Zhen Shi

**Affiliations:** 1 Shandong Provincial Key Laboratory of Animal Cell and Developmental Biology, School of Life Sciences, Shandong University, Qingdao 266237, China; 2 Department of Biopharmacy, School of Biological Sciences and Biotechnology, University of Jinan, Jinan 250022, China

**Keywords:** *Marsupenaeus japonicus*, WSSV, ATP synthase subunit, ATP, antimicrobial peptide genes

## Abstract

Short open reading frames (sORFs) are a newly identified family of genes, and the functions of most sORF genes and their encoded peptides (SEPs) are still unknown. In this study, two ATP synthase subunits were identified in kuruma shrimp (*Marsupenaeus japonicus*) as SEPs, namely *Mj*ATP5I and *Mj*ATP5L. They were widely distributed in all of the tested tissues of shrimp and upregulated in hemocytes and intestines in response to WSSV challenge. The injection of recombinant proteins (r*Mj*ATP5I and r*Mj*ATP5L) increased the expression of *Ie1* and *Vp28*, while the knockdown of *MjATP5I* and *MjATP5L* decreased the expression of *Ie1* and *Vp28*. All of the results suggest that *Mj*ATP5I and *Mj*ATP5L were beneficial for WSSV replication. Further exploration found that *MjATP5I* and *MjATP5L* RNAi significantly improved the shrimp survival rates, reduced ATP production, and upregulated the expression of antimicrobial peptide genes post viral challenge, and the two ATPase subunits and Relish negatively regulated each other. These results reveal that *Mj*ATP5I and *Mj*ATP5L facilitated WSSV duplication by regulating the production of ATP contents and the expression of antimicrobial peptide genes in shrimp.

## 1. Introduction

Kuruma shrimp *Marsupenaeus japonicus*, as one of the important economic animals in aquaculture, are often threatened by diseases caused by viruses, bacteria, fungi, and parasites. It is reported that more than half of shrimp diseases are caused by viruses [1]. In particular, the viral disease in shrimp culture caused by white spot syndrome virus (WSSV) is quite serious and results in significant losses in shrimp aquaculture. However, there are few prophylactic and therapeutic measures to control viral diseases. Understanding the mechanism of shrimp responding to viral infections is essential for controlling these diseases.

Shrimp, as invertebrates, lack adaptive immunity and depend entirely on their innate immunity, which includes humoral immunity and cellular immunity. In humoral immunity, the expression of antimicrobial peptide (AMP) genes is regulated by the Toll pathway, the immune deficiency (IMD) pathway, and the Janus kinase/signal transducer and activator of transcription pathway [2]. Some studies have shown that the expression of AMP genes such as the anti-lipopolysaccharide factors *Alf-b1*, *Alf-c1*, *Alf-c2*, *Alf-d2*, and *Alf-e1* is regulated by transcription factor Relish in the IMD signal pathway [2,3,4]. In shrimp, the AMPs (mainly Alfs) are proved to suppress the WSSV replication and show strong antiviral roles in shrimp immunity [5,6].

In recent years, a new family of small open reading frame genes and their encoded peptides (SEPs) was identified in animals, plants, fungi, and bacteria based on expanded data from next-generation sequencing technologies. They usually encode proteins of less than 100 amino acids in length and are called small open reading frames or short open reading frames (sORFs) [7,8,9]. The sORF family is essentially a hidden genome in the genomes of organisms due to the low molecular mass and the difficulties in screening and identification [7]. It includes thousands of genes, while most of their functions are still unknown [10,11]. So, it is possible to find novel proteins with interesting functions from the sORF family. With the development of related studies, more and more sORFs have been identified to function crucially in some biological processes, such as development, cancer, and innate immunity [8,12,13].

ATP synthase, one of the most important proteins in mitochondria, is the only protein to synthesize ATP in the electron transport chain [14]. In mammals, the ATP synthase is the F_1_F_0_-ATPase, which is located in the inner membrane of mitochondria and forms a hetero-oligomeric complex with a molecular mass of around 650 kDa [15]. The F_1_F_0_-ATP synthase complex fulfils the vital functions in ATP synthesis and produces most of the ATP for cells [16], and is composed of two major parts, the F_0_-ATPase portion and the F_1_-ATPase portion. F_0_-ATPase is fat-soluble, and contains a, b, c, d, e, F_6_, A6L, f, and g subunits [17]. Meanwhile, F_1_-ATPase is water-soluble, and consists of α, β, γ, δ, and ε subunits [18]. In addition, F_1_F_0_-ATP synthase also structurally determines the cristae morphology of mitochondria [16]. Deviations in the structure of ATP synthase and its subunits might lead to various diseases in animals and humans [19,20,21]. The beta subunit of the F_1_-ATP synthase in shrimp, detected only in the granular hemocytes, could bind to WSSV in the immune defense reaction [22,23].

The *ATP5I* gene encodes ATP synthase subunit e in humans, which is one subunit of the mitochondrial F_0_-ATPase complex. ATP5I contains an ATP synthase subunit e domain. Its main function is to interact with the ATP synthase subunit g to promote the ATP synthase forming dimers [24]. It is reported that the antisense strand of *ATP5I* can cause the inhibition of cell growth in human hepatocellular carcinoma [25]. In shrimp *Penaeus vannamei*, the ATP synthase subunit e was screened and identified as a growth-associated marker gene [26]. *ATP5L* gene encodes the ATP synthase subunit g in humans, which is located in the F_0_ portion of the ATPase complex. ATP5L contains an ATP synthase subunit g domain. In addition to its functions in the interaction with ATP synthase subunit e [24], the ATP5L encoded mRNA functions as a direct target of miR-570 to facilitate the ATP loss in human platelet storage [27]. It is reported that the ATP synthase subunit g from yeast mitochondrial F_1_F_0_-ATP synthase is required for the oxidase activity of cytochrome c [28]. In Pacific white shrimp (*P. vannamei*), ATP synthase subunit g was identified and characterized as a candidate gene related to ammonia tolerance [29]. However, the genes encoding for ATP synthase subunits have not been characterized in the innate immunity of invertebrates.

There have been no reports about the ATPase subunits in the innate immunity of shrimp. Therefore, the present study aimed to detect the involvement of ATPase subunits in kuruma shrimp *M. japonicus.* We identified two genes, called *MjATP5I* and *MjATP5L*, in kuruma shrimp, which encoded for ATP synthase subunit e and g, respectively. They both belong to the sORF family, with predicted protein lengths of less than 100 amino acids. The tissue distribution and time course expression profiles of *MjATP5I* and *MjATP5L* were detected at the transcriptional level via quantitative real-time polymerase chain reaction (qPCR) analysis. Recombinant protein injection and the knockdown assay were used to check the potential functions of *Mj*ATP5I and *Mj*ATP5L in vivo. Additionally, the possible mechanisms of action for *Mj*ATP5I and *Mj*ATP5L in the shrimp viral immune reaction were investigated.

## 2. Materials and Methods

### 2.1. Bioinformatics Analysis

The nucleotide sequences of *MjATP5I* and *MjATP5L* were obtained from transcriptome sequencing of kuruma shrimp *M. japonicus* (BGI, Shenzhen, China). The gene sequences of *MjATP5I* and *MjATP5L* were analyzed with BLASTx program in the National Center for Biotechnology Information server (https://blast.ncbi.nlm.nih.gov). The DNA sequences were translated into the amino acid sequences using the translate tool of ExPASy (https://web.expasy.org/translate/), while the isoelectric point and molecular weight were predicted by the compute pI/Mw tool in ExPASy (https://web.expasy.org/compute_pi/). The sequences were aligned by the ClustalW program to construct the neighbor-joining phylogenetic trees by MEGA 5.2 software [30]. Additionally, the functional domains of *Mj*ATP5I and *Mj*ATP5L were detected by the simple module architecture research tool (SMART, http://smart.embl-heidelberg.de/).

### 2.2. Tissue Distribution and Expression Profile Analysis

Healthy kuruma shrimp *M. japonicus* (around 10 g per shrimp) were obtained from a local shrimp farm in Qingdao, Shandong Province, China, and cultured in the recirculating aquaculture system filled with aerated natural seawater at 22 °C. Shrimp were cultured for more than two days until the sample collection and the immune challenge.

Tissues including the hemocytes, heart, hepatopancreas, gills, stomach, and intestines of at least three normal shrimp were collected for the total RNAs extraction with RNAiso Plus (Takara, Dalian, China). Then, they were reversely transcribed into the first strand cDNAs by PrimeScript™ II 1st Strand cDNA Synthesis Kit (Takara, Dalian, China), diluted 20-fold and used as the templates for the distribution analysis by qPCR. The primers *MjATP5I*-RTF with *MjATP5I*-RTR and *MjATP5L*-RTF with *MjATP5L*-RTR (Table 1) for qPCR analysis are listed in Table 1. *β-actin* with the primers *actin*-RTF and *actin*-RTR (Table 1) was used as the internal reference gene. The procedure of qPCR was as follows: 94 °C for 10 min; 40 cycles of 94 °C for 15 s, 60 °C for 60 s, and 76 °C for 2 s; and a melting curve analysis from 65 °C to 95 °C. The tissue distributions of *MjATP5I* and *MjATP5L* were computed using the 2^−ΔΔCT^ method. GraphPad Prism 8.0 (GraphPad Software, San Diego, CA, USA) was used to analyze the data and construct the figures.

For the immune infection, 50 μL (1 × 10^5^ copies) of WSSV was injected into shrimp as the challenged group, while the same volume of sterile PBS (137 mM NaCl, 10 mM Na_2_HPO_4_, 2.7 mM KCl, 1.8 mM KH_2_PO_4_, pH 7.4) was injected into the other shrimp as the control group. Total RNAs were obtained from hemocytes and intestines at 0, 6, 12, 24, 36, and 48 h after the immune challenge. Then, qPCR was carried out to check the expression patterns of *MjATP5I* and *MjATP5L* following the above procedure.

### 2.3. Recombinant Protein Expression and Purification

The full-length open reading frames (ORFs) of *MjATP5I* and *MjATP5L* were amplified with their corresponding expression primer ExF and ExR listed in Table 1. After purification and double restriction enzyme digestion, the DNA fragment for *MjATP5I* was ligated into the *Eco*R I and *Xho* I sites of pET32a(+) vector (Novagen, Madison, WI, USA), while the fragment for *MjATP5L* was ligated into the *Eco*R I and *Not* I sites of pET32a(+) vector using T4 DNA ligase (Thermo Fisher Scientific). Then, after cloning and sequencing to verify the sequence accuracy, the recombinant plasmids *MjATP5I*-pET32a and *MjATP5L*-pET32a were transformed into *Escherichia coli* Rosetta competent cells. Additionally, 0.5 mM isopropyl-1-thio-b-D-galactopyranosid (IPTG) was added to induce the recombinant protein expression at 37 °C for 5 h. The recombinant proteins (r*Mj*ATP5I and r*Mj*ATP5L) were all expressed in the inclusion bodies, which were washed with Buffer A (50 mM Tris-HCl, 5 mM EDTA, pH 8.0) and Buffer B (2 M Urea, 50 mM Tris-HCl, 5 mM EDTA, pH 8.0) twice, and then diluted in Buffer C (8 M Urea, 100 mM Tris-HCl, 10 mM DTT, pH 8.0) at 37 °C for 1 h. After centrifugation at 12,000× *g* for 10 min, the supernatants of the solutes were dialyzed against an at least 50-times greater volume of buffer (100 mM Tris-HCl, 5 mM EDTA, 5 mM cysteine, pH = 8.0) at 4 °C for over 12 h twice. Finally, the supernatants of the dialysates were purified by Ni-nitrilotriacetic acid (Ni-NTA) resin (GE Healthcare, Piscataway, NJ, USA) by means of affinity chromatography following the instructions. The purified proteins were dialyzed against 100 mM Tris-HCl (pH 8.0) and stored at −20 °C for use.

Thioredoxin (Trx) was recombinantly expressed and purified as the control tag protein by transforming the empty pET32a (+) vector into *E. coli* Rosetta competent cells and inducing with IPTG. As expressed in the supernatant, Trx was purified directly with Ni-NTA resin after the ultrasonication of bacteria. The purified Trx protein was dialyzed against 100 mM Tris-HCl (pH 8.0) and cryopreserved at −20 °C for use.

### 2.4. Protein Injection Assay

For the protein injection in vivo, the purified recombinant protein was injected into the shrimp body cavity in vivo. First of all, the recombinant protein (r*Mj*ATP5I or r*Mj*ATP5L) was mixed with WSSV by gently shaking at 4 °C for 1 h. Additionally, the mixture containing 10 μg of recombinant protein with 1 × 10^5^ copies of WSSV was injected into shrimp. Meanwhile, the mixture of Trx (10 μg) with WSSV (1 × 10^5^ copies) was injected into other shrimp simultaneously as the control group.

Additionally, 1 h later, hemocytes were collected for the immunocytochemistry assay to check whether the recombinant protein entered into hemocytes. Briefly, the hemolymph was collected by a 5-mL syringe preloading with the mixture of anticoagulant (450 mM NaCl, 10 mM KCl, 10 mM EDTA, 10 mM HEPES, pH 7.5) and 4% paraformaldehyde in equal proportion. The hemolymph was deposited onto the coated glass slides and sat for at least two hours at room temperature. Then, hemocytes were washed with PBS six times, and treated with 0.2% TritonX-100 at 37 °C for 5 min to enhance the permeability of the hemocyte membrane. After blocking with 3% BSA at 37 °C for half an hour, hemocytes were incubated with mouse anti-His antibody (1:1000 in 3% BSA; ZSGB-BIO, Beijing, China) at 4 °C for at least 12 h, and then hatched with FITC labeled goat anti-mouse IgG(H+L) (1:1000 in 3% BSA; ZSGB-BIO) at 37 °C for 3 h. The nuclei of hemocytes were dyed with 4′,6-diamidino-2-phenylindole (DAPI, 1:1000 diluted in PBS) for 10 min at room temperature. Finally, Olympus BX51 fluorescence microscope (Olympus Corporation, Tokyo, Japan) was used to observe hemocytes and take pictures.

At 48 h after WSSV challenge, total RNAs were extracted from shrimp hemocytes and intestines to reversely transcribe the first-strand cDNAs, which were diluted 20-fold and used as the templates for qPCR to analyze the viral duplication. The expression of WSSV *Ie1* gene and *Vp28* gene was detected by qPCR. The data were analyzed by the 2^−ΔΔCT^ method and processed with GraphPad Prism 8.0 software.

### 2.5. RNA Interference

The primers *MjATP5I*-RNAiF with *MjATP5I*-RNAiR and *MjATP5L*-RNAiF with *MjATP5L*-RNAiR (Table 1) were used to amplify the DNA fragments for the synthesis of double-stranded RNAs (dsRNAs). T7 RNA polymerase (Thermo Fisher Scientific, Waltham, MA, USA) was used to synthesize the dsRNAs for *MjATP5I* and *MjATP5L*. After being purified by chloroform, dsRNAs were diluted to 1 μg/μL with DEPC-treated water. The primer *Gfp*-RNAiF and *Gfp*-RNAiR (Table 1) was used for the amplification of the green fluorescent protein (*Gfp*) nucleotide fragment, which was used to synthesize the *Gfp* dsRNA as the control in the RNA interference (RNAi) assay. Each shrimp was injected with 50 μg of dsRNA. Additionally, 24 h later, another 50 μg of dsRNA was injected into the shrimp. At 24 h after the second dsRNA injection, hemocytes and intestines were isolated for the total RNA extraction. The RNAi effects were detected by qPCR using the primers *MjATP5I*-RTF1 with *MjATP5I*-RTR1 and *MjATP5L*-RTF1 with *MjATP5L*-RTR1 (Table 1). Then, WSSV (1 × 10^5^ copies) was injected into shrimp. Total RNAs were obtained from shrimp hemocytes and intestines at 48 h post WSSV infection. Additionally, the relative expression of WSSV immediate early gene (*Ie1*) and envelope protein 28 gene (*Vp28*) was analyzed by qPCR using the primers listed in Table 1.

The expression of *Relish* and some antimicrobial peptide genes, such as *Alf-b1*, *Alf-c1*, *Alf-c2*, *Alf-d2*, and *Alf-e1* after RNAi of *MjATP5I* and *MjATP5L,* was detected by qPCR using the primers listed in Table 1.

### 2.6. Survival Rate Assay

For the survival rate assay, the shrimp were divided into three groups—the *dsGfp*-injected shrimp, the *dsMjATP5I*-injected shrimp, and the *dsMjATP5L*-injected shrimp. Each group contained at least 30 shrimp. At 24 h after the second dsRNA injection, WSSV (1 × 10^5^ copies) was injected into the shrimp. Then, the number of dead shrimp was recorded every 24 h. The survival rates of each group were statistically analyzed, and the survival curves were constructed using GraphPad Prism 8.0.

### 2.7. ATP Content Detection Assay

After knockdown of *MjATP5I* and *MjATP5L* followed by the WSSV challenge, hemocytes were collected from at least three shrimp as mentioned above. Then, the ATP content in shrimp hemocytes was detected using the ATP content detection kit (BC0300, Solarbio, Beijing, China) following the manufacturers’ instruction. In brief, hemocytes were lysed with the extraction solution, sonicated for 1 min (ice bath, power 20% or 200 W, sonication 2 s and stop 1 s), and centrifuged at the speed of 10,000× *g* for 10 min at 4 °C to separate the pellet and the supernatant. The harvested supernatant was extracted with chloroform to retain the supernatant, which was mixed with reagent I as well as the pre-prepared working solution. Additionally, the absorbance value was measured at 10 s and 3 min 10 s at A_340_ using a spectrophotometer (M200pro, Tecan, Männedorf, Switzerland). The absorbance value of the ATP standard was also determined at the same time. The ATP content in the extracted hemocytes was calculated following the manufacturers’ instructions. The ATP content of the hemocytes from the *Gfp* RNAi shrimp were used as the control. Graphpad Prism 8.0 software was used to analyze results and construct figures.

### 2.8. The Expression Analysis of MjATP5I and MjATP5L after Relish Knockdown

For *Relish* RNAi in shrimp, the primers *Relish*-RNAiF and *Relish*-RNAiR (Table 1) were used to amplify DNA fragment for *Relish* dsRNA, which was synthesized and purified following the above method. After the dsRNA injection, the hemocytes were collected for total RNA extraction and cDNA synthesis. QPCR analysis was performed to check the knockdown effect in hemocytes with primers *Relish*-RTF and *Relish*-RTR (Table 1).

At 24 h after the second dsRNA injection, WSSV (1 × 10^5^ copies) was injected into the shrimp body cavity. Total RNAs were obtained from hemocytes at 48 h post WSSV infection. Then, the relative expression of *MjATP5I* and *MjATP5L* was analyzed by qPCR using primers listed in Table 1. The *Gfp* RNAi shrimp were used as the control.

## 3. Results

### 3.1. Sequence Analysis of MjATP5I and MjATP5L

**Figure 1:**
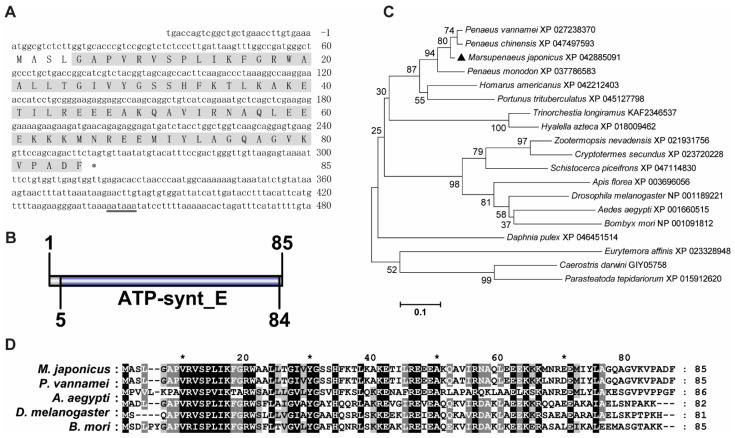
Sequence analysis of *Mj*ATP5I. (**A**) The nucleotide and deduced amino acid sequence of *Mj*ATP5I. The letters with a grey background represent the ATP synthase subunit e domain. The double-underlined nucleotides show the polyadenylation signal region. (**B**) The domain schematics of *Mj*ATP5I. *Mj*ATP5I contained an ATP synthase E domain with 80 amino acids. (**C**) The phylogenetic tree analysis of ATP5Is from different animal species, including *Aedes aegypti*, *Amphibalanus amphitrite*, *Apis florea*, *Bombyx mori*, *Caerostris darwini*, *Cryptotermes secundus*, *Daphnia pulex*, *Drosophila melanogaster*, *Eurytemora affinis*, *Homarus americanus*, *Hyalella azteca*, *Marsupenaeus japonicus*, *Parasteatoda tepidariorum*, *Penaeus chinensis*, *Penaeus monodon*, *Penaeus vannamei, Pollicipes pollicipes*, *Portunus trituberculatus*, *Schistocerca piceifrons*, *Trinorchestia longiramus*, and *Zootermopsis nevadensis*. *Mj*ATP5I is marked by a solid black triangle. All of the amino acid sequences were obtained from GenBank. (**D**) Multiple sequence alignment of ATP5Is from *M. japonicus*, *P. vannamei, A. aegypti*, *D. melanogaster,* and *B. mori*. The conserved sites are labeled by white letters with a black background.

The ORF of *MjATP5I* contained 258 base pairs (bps) of nucleotides, encoding a putative protein of 85 amino acids (Figure 1A). The predicted molecular mass of *Mj*ATP5I was 9.46 kDa with a predicted isoelectric point of 9.57. *Mj*ATP5I contained an ATP synthase subunit e (ATP-synt E) function domain (Figure 1B). From the phylogenetic neighbor-joining (NJ) tree (Figure 1C), we could find that ATP5Is could be divided two groups—one group contained some arthropods, including insects and crustaceans, and the other group contained *Eurytemora affinis*, *Caerostris darwini*, and *Parasteatoda tepidariorum*. *Mj*ATP5I belonged to the crustacean subgroup. A high similarity (96.5%) could be found between *Mj*ATP5I and ATP5I from *P. vannamei* (Figure 1D).

**Figure 2:**
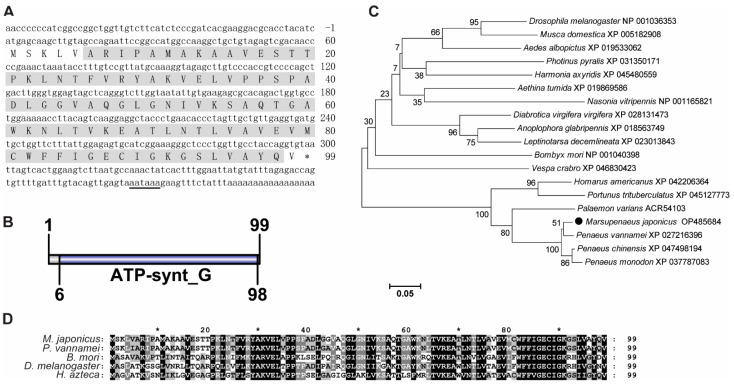
Sequence analysis of *Mj*ATP5L. (**A**) The nucleotide and deduced amino acid sequence of *Mj*ATP5L. The letters with a grey background represent the ATP synthase subunit g domain. The double-underlined nucleotides show the polyadenylation signal region. (**B**) The domain schematics of *Mj*ATP5L. *Mj*ATP5L contained an ATP synthase G domain with 93 amino acids. (**C**) Phylogenetic tree analysis of ATP5Ls from *Aedes albopictus*, *Aethina tumida*, *Anoplophora glabripennis*, *Bombyx mori*, *Diabrotica virgifera virgifera*, *Drosophila melanogaster*, *Harmonia axyridis*, *Homarus americanus*, *Leptinotarsa decemlineata*, *Marsupenaeus japonicus*, *Musca domestica*, *Nasonia vitripennis*, *Palaemon varians*, *Penaeus chinensis*, *Penaeus monodon*, *Penaeus vannamei*, *Photinus pyralis*, *Portunus trituberculatus*, and *Vespa crabro.* ATP5L from *M. japonicus* is labeled by a black solid circle. All of the amino acid sequences were obtained from GenBank. (**D**) Multiple sequence alignment of ATP5L from *M. japonicus*, *P. vannamei, B. mori, D. melanogaster*, and *Hyalella azteca*. The conserved sites are labeled by white letters with a black background.

The ORF of *MjATP5L* had 300 bps, encoding a putative protein of 99 amino acids (Figure 2A). *Mj*ATP5L protein was predicted to have a theoretical molecular mass of 10.47 kDa and a theoretical p*I* value of 9.30. The function domain in *Mj*ATP5L protein was ATP synthase subunit g (ATP-synt G) domain (Figure 2B). The phylogenetic tree showed that these ATP5Ls could be divided into two groups, with one group including insects, and the other group containing crustaceans. *Mj*ATP5L with ATP5Ls from other shrimp were located in the same subgroup of the crustacean group (Figure 2C). The similarity between *Mj*ATP5L and ATP5L from *P. vannamei* was around 97.0% (Figure 2D).

### 3.2. Tissue Distribution and Expression Profiles of MjATP5I and MjATP5L

**Figure 3:**
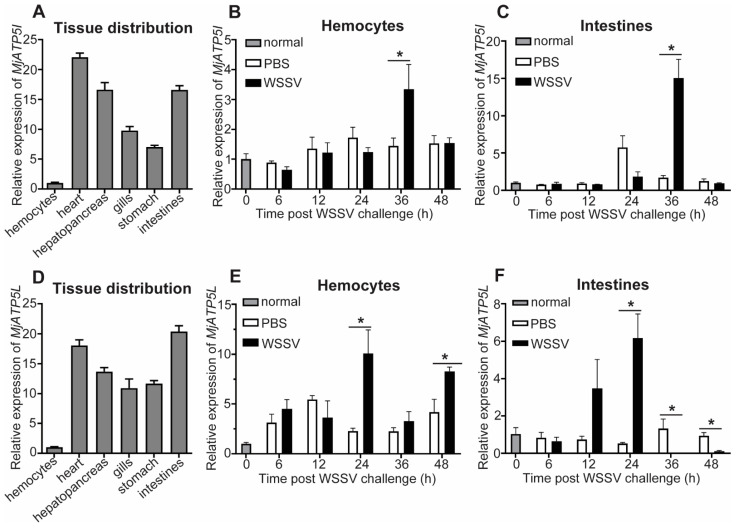
Expression pattern analysis of *MjATP5I* and *MjATP5L*. (**A**) Tissue distribution of *MjATP5I* in tissues such as hemocytes, heart, hepatopancreas, gills, stomach, and intestines. (**B**,**C**) Expression profiles of *MjATP5I* in hemocytes (**B**) and intestines (**C**) post WSSV challenge. (**D**) Tissue distribution of *MjATP5L* in different tissues. (**E**,**F**) Expression profiles of *MjATP5L* in shrimp hemocytes (**E**) and intestines (**F**) after WSSV challenge. The PBS injected shrimp were used as the control. The results are expressed as the mean ± SD and significant differences were analyzed between the PBS-injected group and the WSSV-injected group using Student’s *t*-test. *, *p* < 0.05.

To detect the tissue distribution and expression profiles of *MjATP5I* and *MjATP5L*, qPCR was performed. For the tissue distribution at the transcriptional level, *MjATP5I* could be detected in all six of the tested tissues including hemocytes, heart, hepatopancreas, gills, stomach, and intestines, with the highest expression in heart (Figure 3A). After WSSV challenge, the mRNA expression patterns of *MjATP5I* in the immune related tissues were checked by qPCR. The results show that the relative expression of *MjATP5I* could be upregulated at 36 h post viral immune challenge in hemocytes (Figure 3B) and intestines (Figure 3C).

*MjATP5L* is also distributed in the six selected tissues with the highest expression level in the heart and intestines and the lowest expression level in the hemocytes (Figure 3D). The relative expression of *MjATP5L* in hemocytes could be upregulated significantly at 24 h and 48 h post WSSV infection (Figure 3E) and increased obviously in intestines at 24 h after the viral challenge (Figure 3F).

### 3.3. MjATP5I and MjATP5L Were Beneficial for WSSV Duplication

**Figure 4:**
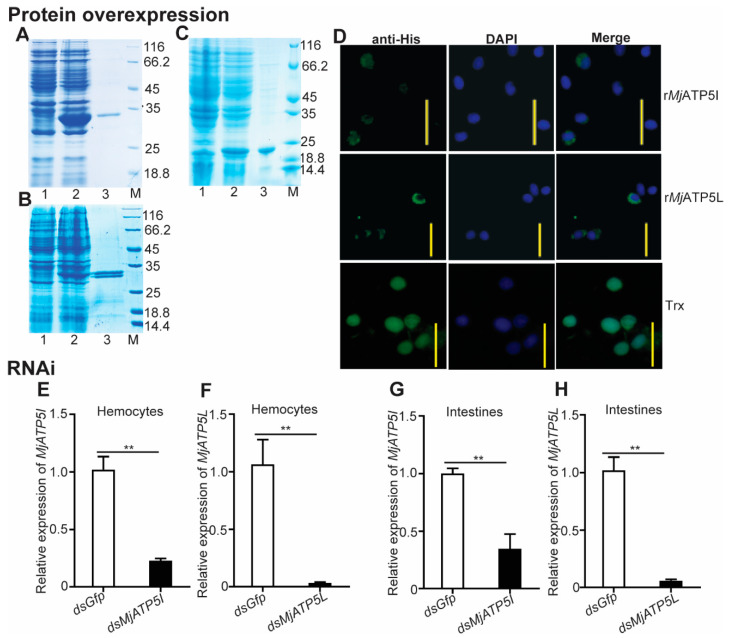
Recombinant protein injection and RNAi effect detection for *Mj*ATP5I and *Mj*ATP5L in shrimp. The recombinant expression and protein purification of *Mj*ATP5I (**A**), *Mj*ATP5L (**B**), and Trx (**C**) in *E. coli*. Lane 1, total protein of *E. coli* with *MjATP5I*-pET32a, *MjATP5L*-pET32a, or empty pET-32a(+) vector without induction; lane 2, total protein of *E. coli* with *MjATP5I*-pET32a, *MjATP5L*-pET32a, or empty pET-32a(+) vector induced with 0.5 mM IPTG; lane 3, the purified r*Mj*ATP5I, r*Mj*ATP5L, or Trx; lane M, the standard protein marker. (**D**) Immunocytochemistry assay to detect the entrance of r*Mj*ATP5I, r*Mj*ATP5L, or Trx into shrimp hemocytes. The recombinant proteins in shrimp hemocytes were indicated by the green fluorescence signals, and the nuclei were stained by DPAI and shown by blue signals. Scale bar = 20 μm. (**E**,**F**) RNA interference effects of *MjATP5I* and *MjATP5L* were detected in hemocytes after RNAi. (**G**,**H**) Interference effects of *MjATP5I* and *MjATP5L* were detected in intestines after RNAi. The results are expressed as the mean ± SD and significant differences were analyzed between the *dsMjATP5I-*injected or *dsMjATP5L*-injected group and the *dsGfp*-injected group using Student’s *t*-test. **, *p* < 0.01.

To check the involvement of *MjATP5I* and *MjATP5L* in WSSV duplication, qPCR was applied for the expression of viral genes after protein injection and RNAi. The predicted molecular masses of r*Mj*ATP5I or r*Mj*ATP5L were 27.44 kDa and 28.45 kDa, respectively, which were close to the molecular masses (around 30 kDa) in SDS-PAGE (Figure 4A,B). In the recombinant protein injection assay, the purified recombinant r*Mj*ATP5I or r*Mj*ATP5L was injected into the shrimp cavity after incubation with WSSV. The effects of recombinant protein injection were checked by immunocytochemistry assay. Considering the size of the tag protein, the purified Trx protein with a molecular mass of 20.40 kDa (Figure 4C), was used as the control to eliminate the effect of the tag protein. The results show that there were green fluorescence signals in the cytoplasm of the shrimp hemocytes post the injection of r*Mj*ATP5I or r*Mj*ATP5L (Figure 4D), while after the injection of Trx tag protein, the green fluorescence signals distributed in the whole hemocytes (Figure 4D), which was different from the distribution of r*Mj*ATP5I or r*Mj*ATP5L. It indicated that r*Mj*ATP5I and r*Mj*ATP5L could enter shrimp hemocytes and located in the cytoplasm to participate in viral immune response.

Meanwhile, after knockdown of *MjATP5I* and *MjATP5L* in shrimp, WSSV was injected into shrimp. Firstly, the interference effect was checked by qPCR. The results show that the relative expression levels of *MjATP5I* and *MjATP5L* decreased obviously in both hemocytes (Figure 4E,F) and intestines (Figure 4G,H) compared to the control group. This demonstrates that *MjATP5I* and *MjATP5L* could be knocked down successfully in shrimp.

**Figure 5:**
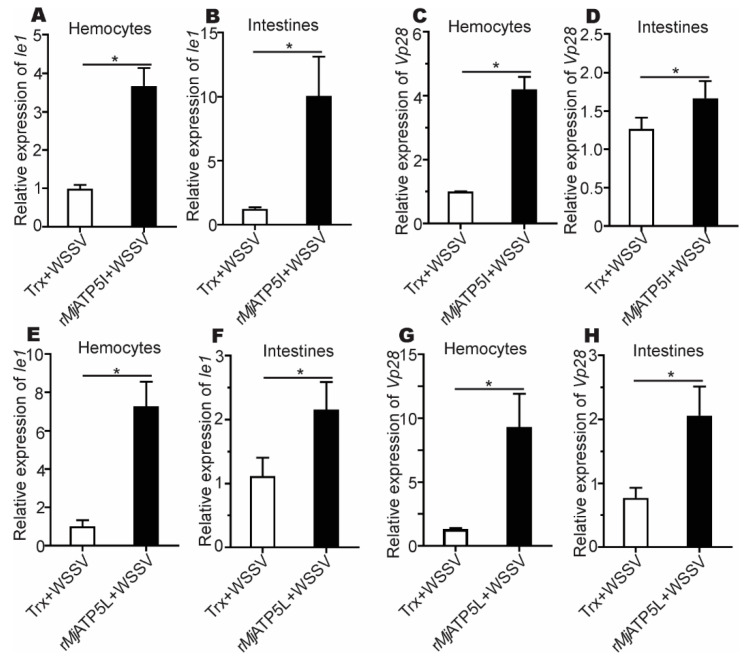
The relative expression of *Ie1* and *Vp28* gene after recombinant protein injection. (**A**–**D**) The expression of *Ie1* (**A**,**B**) and *Vp28* (**C**,**D**) in hemocytes and intestines after r*Mj*ATP5I injection. (**E**–**H**) The expression of *Ie1* (**E**,**F**) and *Vp28* (**G**,**H**) in hemocytes and intestines after r*Mj*ATP5L injection. The results are expressed as the mean ± SD and significant differences were analyzed between r*Mj*ATP5I- or r*Mj*ATP5L-injected group and Trx-injected group using Student’s *t*-test. *, *p* < 0.05.

**Figure 6:**
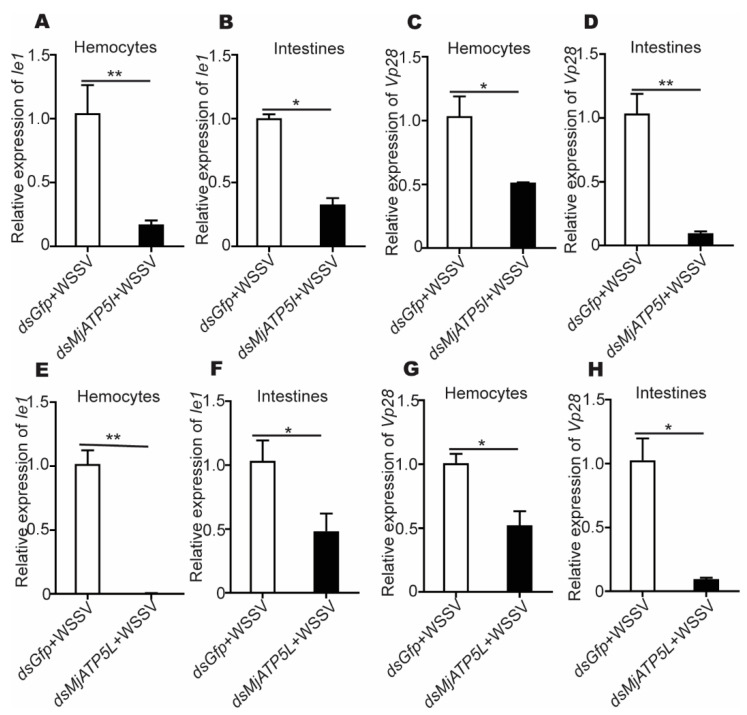
The relative expression of *Ie1* and *Vp28* after RNAi. (**A**–**D**) The relative expression of *Ie1* (**A**,**B**) and *Vp28* (**C**,**D**) in hemocytes and intestines after *MjATP5I* knocking down. (**E**–**H**) The relative expression of *Ie1* (**E**,**F**) and *Vp28* (**G**,**H**) in hemocytes and intestines after *MjATP5L* knocking down. The results are expressed as the mean ± SD and significant differences were analyzed between the *dsMjATP5I*-injected or *dsMjATP5L*-injected group and the *dsGfp*-injected group using Student’s *t*-test. *, *p* < 0.05, **, *p* < 0.01.

After the r*Mj*ATP5I injection, the expression of *Ie1* and *Vp28* increased obviously in hemocytes and intestines (Figure 5A–D), while after *MjATP5I* RNAi followed by viral injection, the relative expression of *Ie1* and *Vp28* decreased significantly in hemocytes and intestines (Figure 6A–D). Similar expression trends could be found in the r*Mj*ATP5L injection (Figure 5E–H) and *dsMjATP5L* injection assay (Figure 6E–H). All the results suggest that *Mj*ATP5I and *Mj*ATP5L are beneficial for the WSSV duplication in kuruma shrimp.

### 3.4. MjATP5I and MjATP5L Influenced the Shrimp Survival Rate and the Production of ATP

**Figure 7:**
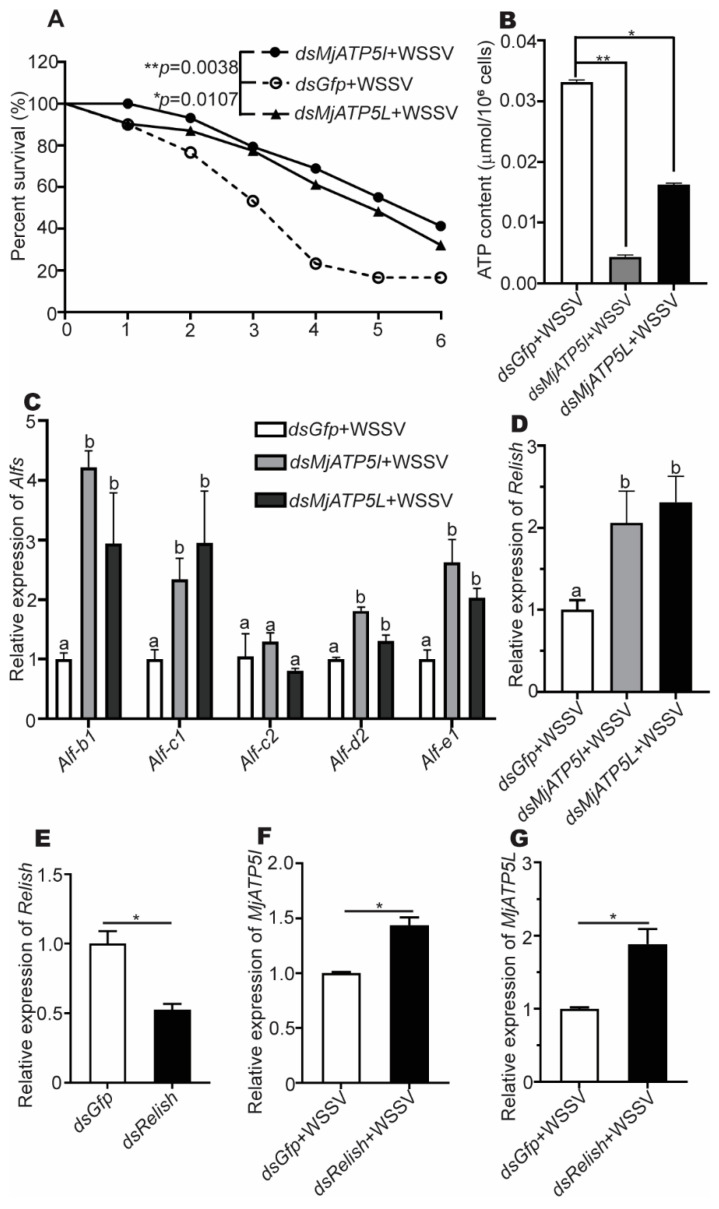
The survival rate, ATP content detection, and the genes relative expression after RNAi and WSSV challenge. (**A**) Survival rates of the *dsMjATP5I*-injected and *dsMjATP5L*-injected shrimp compared with the *dsGfp*-injected shrimp after WSSV infection. The survival rate was calculated to present the survival curves as Kaplan–Meier plots. Differences between the *MjATP5I* RNAi group or *MjATP5L* RNAi group and the *Gfp* RNAi group were analyzed using the Log-rank test in the GraphPad Prism 8.0 program. (**B**) The content of ATP in hemocytes after *MjATP5I* and *MjATP5L* RNAi with WSSV challenge. The *dsGfp*-injected shrimp were used as the control. The results are expressed as the mean ± SD and significant differences were analyzed between the *dsMjATP5I-*injected or *dsMjATP5L*-injected group and the *dsGfp*-injected group using Student’s *t*-test. *, *p* < 0.05, **, *p* < 0.01. (**C**,**D**) Expression of *Alfs* (**C**) and *Relish* (**D**) in hemocytes after *MjATP5I* and *MjATP5L* knockdown. The columns are expressed as the mean ± SD, and the differences were calculated by the ordinary one-way ANOVA with multiple comparisons. The identical letters indicate there are no significant differences between the two groups for the same gene (*p* > 0.05), while different letters indicate significant differences between two groups of the same gene (*p* < 0.05). The *dsGfp*-injected shrimp were used as the control. (**E**) Expression of *Relish* in shrimp hemocytes after *dsRelish* injection. (**F**,**G**) The relative expression of *MjATP5I* (**F**) and *MjATP5L* (**G**) in hemocytes after *Relish* knockdown and WSSV infection. The results are expressed as the mean ± SD and significant differences were analyzed between the *dsMjATP5I-*injected or *dsMjATP5L*-injected group and the *dsGfp*-injected group using Student’s *t*-test. *, *p* < 0.05.

To investigate the influence of the two ATP synthase subunits in shrimp survival, the survival rates were analyzed. The results showed that the survival rates of the *dsMjATP5I*-injected shrimp and the *dsMjATP5L*-injected shrimp were significantly higher than the control group post WSSV challenge (Figure 7A).

To explore the possible mechanism of the two ATP synthase subunits in the shrimp viral immune reaction, the ATP contents in shrimp hemocytes were measured after knockdown of *MjATP5I* and *MjATP5L* with WSSV challenge. We found that the content of ATP decreased dramatically after RNAi of *MjATP5I* and *MjATP5L* in hemocytes (Figure 7B). This revealed that *Mj*ATP5I and *Mj*ATP5L would affect the activity of ATP synthase and the subsequent ATP output of hemocytes in the shrimp defense reaction.

### 3.5. MjATP5I and MjATP5L Could Regulate the Expression of AMP Genes in Shrimp

To test whether *Mj*ATP5I and *Mj*ATP5L regulated the effector molecules of the immune signal pathway, the expression of AMP genes in the downstream of Relish was checked after RNAi and WSSV infection. Figure 7C shows that the expression of *Alf-b1*, *Alf-c1*, *Alf-d2*, *and Alf-e1* was upregulated obviously in the *MjATP5I* and *MjATP5L* RNAi shrimp compared with the *Gfp* RNAi shrimp. However, there were no significant changes in the expression of *Alf-c2*. It was reported that both the Toll-Dorsal and the IMD-Relish pathway could regulate the expression of *Alf-c2*. So, in our assay, the expression of *Alf-c2* might mainly be activated by the Toll-Dorsal pathway. The results demonstrate that some AMP genes under the control of Relish were regulated by *Mj*ATP5I and *Mj*ATP5L.

### 3.6. MjATP5I/MjATP5L and Relish Negatively Regulated Each Other

To investigate the relationship between *Mj*ATP5I*/Mj*ATP5L and Relish, the RNAi assay was performed. After the knockdown of *MjATP5I* and *MjATP5L* and WSSV infection, the relative expression of *Relish* increased significantly in hemocytes from the *dsMjATP5I*-injected or *dsMjATP5L*-injected shrimp compared with the *dsGfp*-injected shrimp (Figure 7D). The results imply that *Mj*ATP5I and *Mj*ATP5L could restrain the expression of Relish.

Meanwhile, after *Relish* RNAi (Figure 7E) and WSSV infection, the expression levels of *MjATP5I* and *MjATP5L* increased remarkably in hemocytes of the *dsRelish*-injected shrimp compared to the control (Figure 7F,G), which demonstrated that Relish repressed the expression of *Mj*ATP5I and *Mj*ATP5L.

Therefore, *Mj*ATP5I/*Mj*ATP5L and Relish negatively regulated each other’s expression in shrimp.

## 4. Discussion

In this study, we identified two ATP synthase subunits, ATP synthase subunit e and ATP synthase subunit g, from kuruma shrimp *M. japonicus*, and explored their possible functions in the viral immune reaction. To our knowledge, this is the first report about the function of the small ATP synthase subunits in shrimp viral immunity.

The two ATP synthase subunits were encoded by *ATP5I* and *ATP5L*, respectively, and defined as *Mj*ATP5I and *Mj*ATP5L in the present study. The amino acid sequences of *Mj*ATP5I and *Mj*ATP5L contained 85 and 99 residues, respectively. They were both newcomers to the sORF family. Recent studies showed that sORF functioned importantly in innate immunity as antimicrobial peptides, in the defense reaction against bacterial infection, in the development process, in the mitochondrial processes, and in the activity regulation of canonical proteins and so on [12,31,32]. Therefore, our identification and exploration of *Mj*ATP5I and *Mj*ATP5L in the viral immune reaction could expand the understanding for the novel functions of the sORF family.

The sequence analysis showed that ATP5I and ATP5L shared high similarities between kuruma shrimp and the other animals, mainly arthropods. It demonstrated that mitochondrial ATP synthase subunits were conservative in evolution. Additionally, from the results of the phylogenetic tree and the multiple sequence alignment, we found that *Mj*ATP5I and *Mj*ATP5L had close relationships with those from *P. vannamei*, which showed a close evolutionary relationship between kuruma shrimp and Pacific white shrimp.

In *M. japonicus*, *MjATP5I* and *MjATP5L* were widely distributed in all six of the examined tissues at the mRNA level. The relative expression of *MjATP5I* and *MjATP5L* could be induced in hemocytes and intestines after WSSV challenge. This indicated that they might participate in the viral immune response of kuruma shrimp. To detect whether shrimp ATP synthase subunits were involved in the viral immunity, we performed recombinant protein injection and RNAi assay, and analyzed the expression patterns of the viral genes after WSSV infection. The results show that the expression of *Ie1* and *Vp28* increased significantly after the injection of recombinant proteins (r*Mj*ATP5I and r*Mj*ATP5L), while the expression of *Ie1* and *Vp28* was downregulated after knockdown of *MjATP5I* and *MjATP5L*. These results indicate that *Mj*ATP5I and *Mj*ATP5L are beneficial for WSSV replication in shrimp.

ATP synthase is one important protein in the mitochondria. The function and mechanism of mitochondrial ATP synthase have already been studied very clearly. In addition to producing ATP for cellular survival, ATP synthase is also related to apoptosis. Pathogenetic variants of the ATP synthase subunit genes can cause fatal human diseases, such as neurodegenerative diseases and motor neuron diseases [33]. It is reported that angiostatin can bind and inhibit F_1_F_0_-ATP synthase to prevent tumor growth and metastasis [34]. Interestingly, besides locating in the inner membrane of the mitochondria, the subunits of ATP synthase were also found to locate in the eukaryotic cell surface and function as the receptor of the cell membrane [35]. Some larger ATP synthase subunits could interact with viruses. For example, the ATP synthase subunit beta was located on the cell surface of hemocytes and gill cells in Pacific white shrimp, *P. vannamei* [22]. It functioned importantly in the antiviral immune reaction to infection with yellow head virus and WSSV in shrimp [36,37,38,39]. In freshwater crayfish, *Pacifastacus leniusculus*, the ATP synthase subunit beta was identified on the plasma membrane of the hematopoietic tissue cells and served as the receptor for the cytokine astakine [35]. In addition to the large subunits, there are some small subunits in the ATP synthase. Most studies have shown that *ATP5I* and *ATP5L* are related to human diseases [21,25,27], and only a few studies reported their involvements in the growth or ammonia tolerance of invertebrates [26,29]. Their exact roles in the viral immunity of shrimp were explored in this study.

After knockdown of *MjATP5I* and *MjATP5L* in shrimp, the shrimp survival rates increased and the content of ATP in hemocytes decreased significantly compared to the control group. It was reported that WSSV would require and consume the host ATP for its replication and infection process [40]. Combined with the above results, we speculated that *Mj*ATP5I and *Mj*ATP5L were related to the ATP synthase activity, provided energy for WSSV replication and infection, and were used for the WSSV duplication and infection in shrimp. Therefore, the two small subunits of ATP synthase were beneficial for the WSSV duplication in shrimp. Knockdown of *MjATP5I* and *MjATP5L* significantly upregulated the expression of Relish and some antimicrobial peptides (*Alf-b1*, *Alf-c1*, *Alf-d2*, and *Alf-e1*). When the shrimp were challenged by WSSV, the expression of *MjATP5I* and *MjATP5L* was upregulated, the expression of Relish was inhibited, and then the expression of some downstream AMP genes decreased subsequently. Finally, the WSSV duplication was facilitated in shrimp. In a word, the ATP synthase subunits would be helpful for the WSSV duplication. The previous study showed that ATPase inhibitor factor 1 was beneficial for WSSV replication in kuruma shrimp *M. japonicus* through inducing the ROS production to activate the NF-κB signal pathway and regulating the expression of Dorsal [41]. Therefore, the mechanism of ATPase inhibitor factor 1 and the small subunits of ATP synthase in the viral immune reaction might be different.

In summary, two novel SEPs, *Mj*ATP5I and *Mj*ATP5L, were identified from kuruma shrimp *M. japonicus. Mj*ATP5I and *Mj*ATP5L can affect the activity of ATP synthase and the expression of the transcriptional factor Relish with the downstream AMP genes, and then facilitate the WSSV replication in shrimp.

## Figures and Tables

**Table 1:** Primers used in this study.

Primer	Gene Target and GenBank Accession No.	Sequence (5′–3′)
*MjATP5I*-RTF	*MjATP5I* XP_042885091	gccacttcaagaccctaa
*MjATP5I*-RTR		tctgctggaaccttcact
*MjATP5L*-RTF	*MjATP5L* OP485684	ccccgaaactaaatacct
*MjATP5L*-RTR		gaaccagcacatcacctc
*MjATP5I*-RNAiF		taatacgactcactataggctcccttgattaagtttgg
*MjATP5I*-RNAiR		taatacgactcactataggcattgggttaggtgtctc
*MjATP5L*-RNAiF		taatacgactcactataggccgttatgcaaaggtagagc
*MjATP5L*-RNAiR		taatacgactcactataggtggtaggcaaccagggag
*MjATP5I*-RTF1		gccacttcaagaccctaa
*MjATP5I*-RTR1		tctgctggaaccttcact
*MjATP5L*-RTF1		ccccgaaactaaatacct
*MjATP5L*-RTR1		gaaacttctttatttactca
*Gfp*-RNAiF	*Gfp* MK371210.1	gcgtaatacgactcactataggtggtcccaattctcgtggaac
*Gfp*-RNAiR		gcgtaatacgactcactataggcttgaagttgaccttgatgcc
*Relish*-RNAiF	*Relish* MN607236	taatacgactcactatagggtcggaggaaggttctcacggc
*Relish*-RNAiR		taatacgactcactatagggtctcctttcctgaccttgtctgtg
*Relish*-RTF		acgcagaagaatgctgattg
*Relish*-RTR		attcccccaccaacagc
*Mj*ATP5I-ExF		tacgaattcatggcgtctcttggtgcaccc
*Mj*ATP5I-ExR		tacctcgagctagaagtctgctggaacctt
*Mj*ATP5L-ExF		tacgaattcatgagcaagcttgtagccaga
*Mj*ATP5L-ExR		tacgcggccgcttacacctggtaggcaaccag
*Ie1*-RTF	*Ie1*	gactctacaaatctctttgcca
*Ie1*-RTR	926915	ctacctttgcaccaattgctag
*Vp28*-RTF	*Vp28*	agctccaacacctcctccttca
*Vp28*-RTR	927064	ttactcggtctcagtgccaga
*Alf-b1*-RT-F	*Alf-b1* KY627759	cggtggtggccctggtggcactcttcg
*Alf-b1*-RTR		gactggctgcgtgtgctggcttcccctc
*Alf-c1*-RTF	*Alf-c1* KU213608	cgcttcaagggtcggatgtg
*Alf-c1*-RTR		cgagcctcttcctccgtgatg
*Alf-c2*-RTF	*Alf-c2* KU160498	tcctggtggtggcagtggct
*Alf-c2*-RTR		tgcgggtctcggcttctcct
*Alf-d2*-RTF	*Alf-d2* MT977630	cgcaggcttatggaggac
*Alf-d2*-RT-R		aggtgacagtgccgagga
*Alf-e1*-RTF	*Alf-e1* KY627760	tcctaaccacgcagtgctttgctaatg
*Alf-e1*-RTR		gcttttcggatttgccttcgatgtttg
*actin-RTF*	*β-actin* GU645235	cagccttccttcctgggtatgg
*actin-RTR*		gagggagcgagggcagtgatt

## Data Availability

Not applicable.
